# Longitudinal association between changes in resting-state network connectivity and cognition trajectories: The moderation role of a healthy diet

**DOI:** 10.3389/fnhum.2022.1043423

**Published:** 2023-01-19

**Authors:** Alexandra M. Gaynor, Eleanna Varangis, Suhang Song, Yunglin Gazes, Christian Habeck, Yaakov Stern, Yian Gu

**Affiliations:** ^1^Taub Institute for Research on Alzheimer’s Disease and the Aging Brain, Columbia University, New York, NY, United States; ^2^Cognitive Neuroscience Division, Department of Neurology, Columbia University, New York, NY, United States; ^3^Gertrude H. Sergievsky Center, Columbia University, New York, NY, United States; ^4^Department of Psychiatry, Columbia University, New York, NY, United States; ^5^Department of Epidemiology, Joseph P. Mailman School of Public Health, Columbia University, New York, NY, United States

**Keywords:** Mediterranean diet, resting-state connectivity, cognitive performance, moderation, fMRI

## Abstract

**Introduction:**

Healthy diet has been shown to alter brain structure and function and improve cognitive performance, and prior work from our group showed that Mediterranean diet (MeDi) moderates the effect of between-network resting-state functional connectivity (rsFC) on cognitive function in a cross-sectional sample of healthy adults. The current study aimed to expand on this previous work by testing whether MeDi moderates the effects of changes in between- and within-network rsFC on changes in cognitive performance over an average of 5 years.

**Methods:**

At baseline and 5-year follow up, 124 adults aged 20–80 years underwent resting state fMRI to measure connectivity within and between 10 pre-defined networks, and completed six cognitive tasks to measure each of four cognitive reference abilities (RAs): fluid reasoning (FLUID), episodic memory, processing speed and attention, and vocabulary. Participants were categorized into low, moderate, and high MeDi groups based on food frequency questionnaires (FFQs). Multivariable linear regressions were used to test relationships between MeDi, change in within- and between-network rsFC, and change in cognitive function.

**Results:**

Results showed that MeDi group significantly moderated the effects of change in overall between-network and within-network rsFC on change in memory performance. Exploratory analyses on individual networks revealed that interactions between MeDi and between-network rsFC were significant for nearly all individual networks, whereas the moderating effect of MeDi on the relationship between within-network rsFC change and memory change was limited to a subset of specific functional networks.

**Discussion:**

These findings suggest healthy diet may protect cognitive function by attenuating the negative effects of changes in connectivity over time. Further research is warranted to understand the mechanisms by which MeDi exerts its neuroprotective effects over the lifespan.

## Introduction

A growing body of research suggests modifiable lifestyle factors, including healthy diet, may protect cognitive function against negative effects of age- and disease-related changes in brain structure and function. Several healthy dietary patterns, as well as individual food types and nutrients comprising healthy dietary patterns, have been shown to benefit brain and cognitive health (Scarmeas et al., 2009; Gu and Scarmeas, 2011; Bourassa et al., 2016; Castelli et al., 2018). One of the most widely studied examples of a healthy diet is the Mediterranean Diet (MeDi), which has been associated with lower incidence of Alzheimer’s disease (AD) (Scarmeas et al., 2006) decreased risk for mild cognitive impairment (MCI) (Scarmeas et al., 2009), and slower cognitive decline (Scarmeas et al., 2006; Tangney et al., 2011; Valls-Pedret et al., 2015).

The biological mechanisms by which healthy diet contributes to cognitive function is not well understood, but some studies have suggested components of a healthy diet may alter cytokine activity and apoptotic pathways to reduce inflammation and oxidative stress (Siervo et al., 2021), and healthy diet has been associated with telomere length, a biomarker that is predictive of neurodegenerative disease (Canudas et al., 2020). Healthy diet may also indirectly benefit neurocognitive health by lowering risk of chronic diseases known to impact cognitive function, such as cardiovascular disease, hypertension, and diabetes (Wu and Sun, 2017). There is limited research on the relationship between diet and brain imaging biomarkers, but the small number of existing studies have suggested MeDi is associated with structural and functional measures of brain health. For instance, studies have shown that greater adherence to MeDi, or intake of components of MeDi, is positively associated with structural brain measures, including greater brain volume (Titova et al., 2013; Gu et al., 2015), cortical thickness (Mosconi et al., 2018), and structural connectivity (Gu et al., 2016; Rodrigues et al., 2020). Furthermore, recent work from our group demonstrated that MeDi may protect against white matter hyperintensities burden increase over time, suggesting healthy diet contributes to cognitive resilience by reducing brain impairment (Song et al., 2022). Moreover, MeDi may reduce the risk of neurodegeneration, as evidenced by its association with slower decline in gray matter (Luciano et al., 2017), lower white matter hyperintensity burden (Gardener et al., 2012), better preserved structural connectivity over time (Pelletier et al., 2015), and lower beta amyloid burden and accumulation over time (Merrill et al., 2016; Rainey-Smith et al., 2018).

Despite growing evidence that MeDi may be beneficial for structural brain health, few studies have examined the effects of MeDi on neuroimaging measures of brain function. There is evidence that between-network resting-state functional connectivity (rsFC) among large-scale networks at rest tends to increase with age, reflecting reduced segregation and specialization of networks underlying cognitive functions (Geerligs et al., 2014; Varangis et al., 2019a; He et al., 2020; Malagurski et al., 2020). Conversely, within-network connectivity, measured by correlations between regions within a given resting state network, tends to decrease with age, reflecting reduced coherence and communication within functional networks. rsFC is thought to play a role in cognition *via* experience-driven changes in connectivity, wherein repeated co-activation among regions engaged during cognitive tasks leads to changes in functional networks at rest (Stevens and Spreng, 2014), and increases in connectivity between resting state networks, and decreases in connectivity within networks, have been associated with poorer cognitive performance in both cross-sectional (Sala-Llonch et al., 2012; Geerligs et al., 2014; King et al., 2018; Varangis et al., 2019b) and longitudinal (Ng et al., 2016; Malagurski et al., 2020) studies, consistent with evidence that underlying resting-state connectivity is crucial to task-related processing during cognitive tasks (Ito et al., 2017).

In the first study to date to investigate the relationship between MeDi, functional connectivity, and cognitive performance, work from our group recently showed that MeDi adherence moderated the cross-sectional relationship between rsFC and cognitive performance: for individuals less adherent to MeDi, lower between-network rsFC was associated with poorer fluid reasoning (FLUID) performance, but the relationship between rsFC and cognition was attenuated in those with moderate and high MeDi adherence (Gaynor et al., 2022). These findings suggest individuals who are more closely following MeDi may have better cognitive abilities in the face of functional brain profiles that are typically associated with lower cognitive performance. However, no studies have investigated the role of MeDi in moderating the relationship between changes in brain function and changes in cognitive performance over time, leaving a gap in knowledge about the protective effect of diet in age-related neurological and cognitive decline. Moreover, the few studies testing the effect of diet on brain biomarkers or cognitive function have focused primarily on older adults; as such, it remains unknown whether healthy diet moderates brain-cognition relationships in younger or middle age, which is critical to understanding at what point in the lifespan adopting a healthy diet may provide neurocognitive benefits.

The present study aimed to (1) test the relationship between MeDi and longitudinal change of between- and within-network rsFC, and (2) test whether MeDi moderates the longitudinal association between rsFC and cognitive function in a sample of healthy adults across the lifespan. Because diet has been shown to impact whole-brain measures of brain structure and function (Gu et al., 2015; Staubo et al., 2017; Gaynor et al., 2022), as well as multiple cognitive functions (Gu and Scarmeas, 2011; Gardener et al., 2015), we tested the effect of MeDi on changes in overall within- and between-network rsFC and their association with four cognitive domains. We hypothesized that individuals who are more adherent to MeDi would demonstrate (1) less mean change in overall between- and within-network rsFC over 5 years, and (2) a weaker association between change in rsFC and change in cognitive performance, reflecting a potential protective effect of MeDi against cognitive sequelae of changes in rsFC.

## Materials and methods

### Participants

The current study included adults aged 20–80 years, who completed the baseline and follow-up visits for two ongoing studies: the Reference Ability Neural Network (RANN) study (Stern et al., 2014) and the Cognitive Reserve study (Stern et al., 2018), both conducted at Columbia University Irving Medical Center with similar recruitment and research procedures. At baseline, 562 participants were enrolled across both studies. As of January 2020, 254 participants returned for a follow-up visit after an average of 5 years following their baseline visit. For the current analyses, participants were excluded if they did not have diet questionnaire data (*N* = 25), or baseline and follow-up rsFC data (*N* = 55); of the 174 remaining participants, 49 were excluded due to missing diffusion tensor imaging fractional anisotropy (DTI FA) data, and 1 due to missing white matter hyperintensity (WMH) data, resulting in a final sample size of *N* = 124.

All participants were native English speakers, right-handed, free of MRI contraindications, and read at a fourth-grade level or above. No participants had any psychological or medical conditions that could affect cognitive function, and no older adults met criteria for dementia or MCI. All participants provided informed consent, and all methods were approved by and performed in accordance with the relevant guidelines and regulations of the Institutional Review Board of the College of Physicians and Surgeons of Columbia University.

### Dietary assessment

Habitual dietary information in the past year was self-reported at the baseline visit using a 61-item version of Willett’s semi-quantitative food frequency questionnaire (FFQ) (Willett et al., 1985). Frequency of consumption per month was calculated for each of 11 food categories and assigned frequency categories from 0 to 5. For components more similar to those in MeDi (non-refined cereals, potatoes, fruits, vegetables, legumes, fish, olive oil), increasing scores corresponded to increased intake; components less characteristic of MeDi (poultry, red meat, full fat dairy products) were reverse-scored, such that higher scores corresponded to lower frequency. For alcohol, no consumption and more than 60 servings per month were both assigned a score of 0; scores 5–1 were assigned for 1–2, 3–4, 5–14, 15–30, and 31–60 servings per month, respectively. Total MeDi score reflects the sum of scores for all food categories, resulting in scores from 0 to 55, with higher score indicating a dietary pattern more characteristic of MeDi (Panagiotakos et al., 2006). Continuous MeDi scores were categorized into three groups (low, moderate, high) based on tertiles of total scores.

### Cognitive function

Cognitive function was measured using performance on six cognitive tasks for each of four reference abilities (RAs): FLUID, processing speed and attention (SPEED), memory (MEMORY), and vocabulary (VOCAB). As described in previous work from our group (Stern et al., 2014; Habeck et al., 2016), each ability was estimated by three neuropsychological out-of-scanner tests and three computerized in-scanner tasks, at both baseline and follow-up visits. Cognitive change scores were calculated as follow-up scores minus baseline scores. FLUID composite score was measured using WAIS-III Block design, WAIS-III Letter–Number Sequencing, and WAIS-III Matrix Reasoning tests (out of scanner), and proportion of correct trials from Paper Folding, Matrix Reasoning, and Letter Sets tasks (in scanner). SPEED score was estimated using WAIS-R Digit Symbol subtest, Trail Making test Part A, and Stroop Color naming subtest (out of scanner), and mean reaction times on correct trials for Digit Symbol, Letter Comparison, and Pattern Comparison tasks (in scanner). MEMORY was measured by the Selective Reminding Task subtests of long-term storage, continuous long-term retrieval, and words recalled on last trial (out of scanner), and Logical Memory, Word Order Recognition, and Paired Associate tasks (in scanner). VOCAB composite score was calculated based on WAIS-III vocab subtest, Wechsler Test of Adult Reading, and American National Adult Reading Test (NART) (out of scanner), and proportion correct on Synonyms, Antonyms, and Picture Naming tasks (in scanner). For more details on cognitive task parameters, please see Stern et al. (2014). Performance on each task was z-scored relative to the mean and standard deviation of the entire cohort. Z-scores for tasks within each cognitive domain were then averaged to produce four reference ability z-scores for each participant.

### MRI procedures and rsFC assessment

Magnetic resonance images were acquired on a 3T Philips Achieva Magnet, over two 2-h sessions. Participants underwent 9.5 min of fMRI blood oxygen level-dependent (BOLD) resting state scans. T1-weighted whole brain images were acquired for each subject using magnetization-prepared rapid gradient-echo (MPRAGE) sequence. Diffusion tensor imaging (DTI) and fluid-attenuated inversion recovery (FLAIR) scans were also acquired. Scanning parameters can be found in Supplementary Table 1.

Resting-state fMRI pre-processing included discarding the first three volumes, slice-timing correction and motion correction performed in FSL (Jenkinson et al., 2012), frame-wise displacement (FWD) calculation, and volume replacement for contaminated volumes (scrubbing). Data were temporally filtered by passing motion-corrected signals through a band-pass filter with the cutoff frequencies of 0.01 and 0.09 Hz using flsmaths-bptf (Jenkinson et al., 2012). Processed data were residualized by regressing out FWD, root mean square difference of BOLD signal, left and right hemisphere white-matter, and lateral-ventricular signals. T1 image segmentation was performed using FreeSurfer software and visually inspected for inaccuracies.

Diffusion tensor imaging data were pre-processed through standard pre-processing pipeline including eddy- and motion correction using FSL. TRACULA (Yendiki et al., 2011) toolbox, distributed as part of FreeSurfer (v5.2.0), was used to derive mean FA for 18 major white matter tracts, and averaged to produce a single FA variable for each participant. WMH was derived from FLAIR imaging using the Lesion Segmentation Toolbox for Statistical Parametric Mapping, images were visually inspected and manually corrected for errors, and WMH volumes were log transformed prior to analysis. Mean cortical thickness and total brain volume were obtained using T1-weighted MPRAGE scans, and standard FreeSurfer parcelation schemes were used to calculate mean thickness and volume based on 68 cortical regions of interest (Desikan et al., 2006).

For within- and between-network rsFC, 264 ROIs defined by Power et al. (2011) were transferred to native space *via* inverse transformation based on the non-linear registration of each subject’s structural scan to MNI template using ANTS software package. Ten mm radius spherical masks were generated for each coordinate and intersected with the FreeSurfer gray matter mask to obtain the gray matter-registered ROI masks for each ROI. An intermodal, intra-subject, rigid-body registration of the fMRI reference image and T1 scan was then performed using FLIRT with 6 degrees of freedom, normalized mutual information as the cost function (Jenkinson and Smith, 2001), in order to transfer ROI masks from T1 space to fMRI space. These transferred ROI masks were used to average all voxels within each mask to obtain a single fMRI time-series for each of the 264 ROIs. Time-series data from each ROI were used to generate correlation matrices among all ROIs (264 × 264 ROIs) and were then z-transformed to generate normalized correlation matrices for each participant. ROIs with centers located within 20 mm of one another were set to zero as per Power et al. (2011). All negative correlation values were excluded from mean correlation computations, and average positive correlations were computed between and within all networks of interest. Given considerable ambiguity in interpretation of negative correlations (Murphy et al., 2009; Chai et al., 2012), the current analyses included only positive correlations in each participant’s correlation matrices (Chan et al., 2014).

Consistent with our previous work examining the role of MeDi in rsFC (Gaynor et al., 2022), ROIs were labeled based on the Power et al. (2011) network assignments (Power et al., 2011), and networks of interest for the current analyses included: Somatomotor Hand (Hand; 30 ROIs), Visual (Vis; 31 ROIs), Somatomotor Mouth (Mouth; 5 ROIs), Auditory (Aud; 13 ROIs), Default Mode (DMN; 58 ROIs), Salience (Sal; 18 ROIs), Cingulo-Opercular (CO; 14 ROIs), Frontoparietal (FP; 25 ROIs), Dorsal Attention (DAN; 11 ROIs), and Ventral Attention (VAN; 9 ROIs). The primary functional connectivity outcomes in the present study were the longitudinal changes of (1) the mean between-network correlation across all 10 networks (1 value reflecting overall mean between-network connectivity, calculated as the mean of all 45 between-network connectivity values), and (2) mean within-network correlations of the 10 within-network values (1 value, calculated as the mean of the 10 within-network connectivity values).

### Data analyses

Descriptive statistics were used to present participant characteristics including age, education, sex, race, ethnicity, and IQ scores, and chi-squared tests and one-way analyses of variance were used to test differences across MeDi groups.

Multivariable linear regression models were used to examine the relationship between MeDi group and the changes in overall between-network and within-network rsFC. In order to test whether the associations between rsFC change and cognitive change differed based on level of MeDi adherence, i.e., moderated by level of MeDi adherence, we conducted moderation analyses in which we added into the regression models the multiplicative interaction term “MeDi group X overall between-network rsFC change” or “MeDi group X overall within-network rsFC change.” A significant interaction term would indicate the associations between rsFC change and cognitive change differ based on level of MeDi adherence. All models were adjusted for age, sex, race/ethnicity, education, NART IQ (Nelson, 1982), total caloric intake, and percentage of motion artifact removed. To examine effects specific to functional connectivity independent of structural brain measures shown to be associated with MeDi and with cognitive function, we also controlled for mean cortical thickness, total brain volume, white matter hyperintensity burden, and mean FA (Gardener et al., 2012; Gu et al., 2015; Cabeza et al., 2016; Song et al., 2022).

For between-network rsFC, exploratory analyses were then conducted to examine the 10 individual network-based connectivity, i.e., the mean correlation between ROIs in each network and ROIs in the nine other networks, in order to probe network-specific effects, producing 10 values reflecting mean between-network connectivity from each of the 10 networks. Similarly, for within-network rsFC, exploratory analyses were conducted to examine connectivity among all ROIs within each of the networks, producing 10 values reflecting mean connectivity within each network. Further exploratory analyses for both between-network and within-network rsFC included stratifying the above moderation analyses by age groups based on tertiles: [younger adult (*N* = 44, *M* = 31.93 years, SD = 7.17 years, range = 22–44 years); middle-aged adult (*N* = 44, *M* = 58.27, SD = 6.15, range = 46–65); older adult (*N* = 36, *M* = 69.47, SD = 3.05, range = 66–78)].

All analyses were conducted in SPSS (version 27.0). Significance was indicated by two-sided *p* < 0.05, with the exception of *p*-values of interaction terms with a significance level of *p* < 0.10.

## Results

### Descriptive analyses

Participants were on average 52.18 (SD = 16.77, range 22–78) years old, and had a mean of 16.20 (SD = 2.33, range 12–22) years of education. About 49% were female, and 59.7, 23.4, and 16.9% of participants identified as non-Hispanic white, non-Hispanic black, and other race/ethnicity, respectively. Mean NART IQ score was 117.64 (SD = 7.94, range 94.16–130.88).

One-way analyses of variance showed there were no significant differences between MeDi groups in baseline measures of mean age [*F*_(2,123)_ = 0.752, *p* = 0.474], years of education [*F*_(2,123)_ = 1.291, *p* = 0.279], NART IQ [*F*_(2,123)_ = 0.184, *p* = 0.832], cortical thickness [*F*_(2,123)_ = 0.128, *p* = 0.880], total brain volume [*F*_(2,123)_ = 0.297, *p* = 0.743], WMH [*F*_(2,123)_ = 0.212, *p* = 0.810], DTI FA [*F*_(2,123)_ = 1.457, *p* = 0.237], or baseline total scrub percentage [*F*_(2,123)_ = 0.189, *p* = 0.828]. There was a significant difference in caloric intake among MeDi groups [*F*_(2,123)_ = 3.365, *p* = 0.038], with greater caloric intake in the high MeDi group. Chi-squared tests showed MeDi groups did not differ by race (*X*^2^ = 1.018, *p* = 0.907), but females were more likely to have middle and high tertile of MeDi than males (*X*^2^ = 8.043, *p* = 0.018).

At 5-year follow-up, overall between-network rsFC was nominally but insignificantly lower (*M* = −0.012, SD = 0.072) and within-network rsFC was nominally but insignificantly higher (*M* = −0.014, SD = 0.079) as compared to at baseline visit. Performance on FLUID, MEMORY, AND SPEED also decreased over time, with an average change of (*M* = −0.148, SD = 0.117), (*M* = −0.144, SD = 0.676), and (*M* = −0.178, SD = 0.151), respectively. Mean VOCAB performance increased with an average change of *M* = 0.065 (SD = 0.159). One-way analyses of variance showed no differences across three MeDi groups in change in overall between-network rsFC [*F*_(2,123)_ = 1.890, *p* = 0.156] change in overall within-network rsFC [*F*_(2,123)_ = 1.014, *p* = 0.366], or change in performance on the four RAs: FLUID [*F*_(2,124)_ = 0.099, *p* = 0.906], MEMORY [*F*_(2,124)_ = 0.192, *p* = 0.825], VOCAB [*F*_(2,124)_ = 0.701, *p* = 0.498], SPEED [*F*_(2,124)_ = 0.643, *p* = 0.527] (Table 1).

**TABLE 1:** Participant characteristics by MeDi group.

	Low MeDi	Moderate MeDi	High MeDi	Sig. (*p*)
*N*	43	38	43	
Age (years)	49.67 (17.62)	53.92 (15.74	53.14 (16.88)	0.474
Education (years)	16.40 (2.34)	16.50 (2.61)	15.74 (2.04)	0.279
% Female	32.6	63.2	53.5	0.018*
% Non-Hispanic white	58.1	57.9	62.8	0.907^a^
% Non-Hispanic black	20.9	26.3	23.3	–
% Other race/ethnicity	20.9	15.8	14.0	–
NART IQ	117.47 (8.35)	118.28 (8.57)	117.25 (7.06)	0.832
Caloric intake	1230.67 (520.75)	1295.84 (533.35)	1542.36 (681.97)	0.038*
Mean cortical thickness	2.56 (0.12)	2.55 (0.10)	2.56 (0.14)	0.880
Total brain volume	−2212.19 (50111.39)	4260.79 (48544.18)	5257.72 (46677.48)	0.743
WMH DTI FA Scrub% Overall BN rsFC Δ Overall WN rsFC Δ FLUID Δ MEMORY Δ VOCAB Δ SPEED Δ	1315.56 (3635.93) 0.448 (0.020) 4.92 (6.71) −0.015 (0.056) −0.021 (0.070) −0.141 (0.118) −0.102 (0.757) 0.064 (0.172) −0.160 (0.150)	882.79 (2028.84) 0.440 (0.021) 5.96 (9.17) 0.006 (0.098) −0.002 (0.098) −0.151 (0.104) −0.137 (0.766) 0.088 (0.159) −0.198 (0.131)	1415.16 (5138.51) 0.444 (0.020) 5.35 (6.91) −0.024 (0.055) −0.020 (0.067) −0.151 (0.130) −0.192 (0.495) 0.045 (0.147) −0.179 (0.167)	0.810 0.237 0.828 0.156 0.366 0.906 0.825 0.498 0.527

*p < 0.01.

*^a^p*-value reflects chi-square test of all 3 race/ethnicity categories by MeDi group. Mean (SD) reported for continuous outcomes, percentages reported for categorical outcomes.

*p*-values reflect differences between MeDi groups based on one-way ANOVAs and Pearson’s chi-square tests.

WMH, white matter hyperintensities; DTI FA, fractional anisotropy; Scrub%, percentage of motion artifact removed; BN, between-network; WN, within-network; Δ, change from baseline to followup; FLUID, fluid reasoning; MEMORY, episodic memory; VOCAB, vocabulary; SPEED, perceptual speed.

### Association of MeDi with change in overall rsFC and with change in cognitive performance

Analyses using multivariable linear regressions controlling for covariates described above revealed a marginally significant effect of MeDi group on overall between-network rsFC change among all 10 networks (*p* = 0.096), wherein the moderate MeDi group had greater increase in overall between-network rsFC than the high MeDi group (*B* = 0.033 [0.002, 0.063], *p* = 0.035). MeDi group was not associated with overall within-network rsFC change (*p* = 0.275). MeDi group was not a significant predictor of change in MEMORY (*p* = 0.366), FLUID (*p* = 0.956), SPEED (*p* = 0.264), or VOCAB (*p* = 0.128) performance from baseline to follow-up visits.

### Interaction between MeDi and change in overall between-network rsFC on change in cognition

**FIGURE 1:**
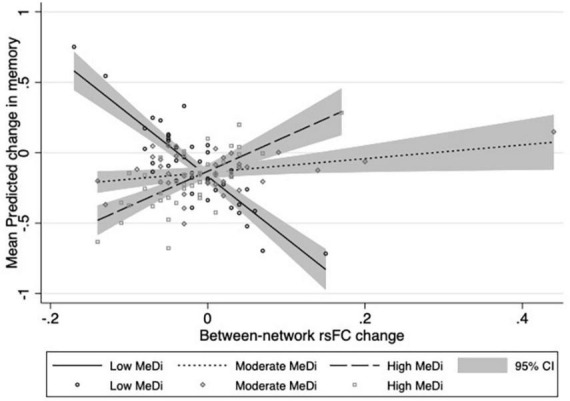
Relationship between change in overall between-network rsFC and change in memory performance by MeDi group. Results of multivariable linear regressions modeling interaction between MeDi group and change in overall between-network rsFC on change in memory performance. Models adjusted for effects of age, gender, race, IQ, caloric intake, mean cortical thickness, diffusion tensor imaging fractional anisotropy, white matter hyperintensities, total brain volume, and scrub percentage. CI, confidence intervals.

For MEMORY, we found a significant interaction between MeDi group and overall between-network rsFC change on change in performance (*p-interaction* = 0.033): relative to the low MeDi group, the association of the between-network rsFC change with the change in MEMORY was weaker in the moderate MeDi group (*p-interaction* = 0.053) and high MeDi group (*p-interaction* = 0.010) (Table 2 and Figure 1), suggesting MeDi adherence moderates the effect of change in between-network rsFC on change in memory performance. Stratified analyses showed that change in overall between-network rsFC had a significant negative effect on change in MEMORY (i.e., increase in between-network rsFC was associated with larger decline in MEMORY or smaller increase in MEMORY) in the low MeDi group (*B* = −4.397 [−8.269, −0.524], *p* = 0.026), with no significant effect of change in between-network rsFC on MEMORY change for the moderate MeDi group. For the high MeDi group, there was a significant positive effect of change in between-network rsFC on change in MEMORY (*B* = 3.287 [0.823, 5.750], *p* = 0.009) (Table 3). MeDi did not moderate the relationship between change in overall between-network rsFC and change in FLUID (*p-interaction* = 0.713), SPEED (*p-interaction* = 0.748), or VOCAB (*p-interaction* = 0.398) (Table 2).

**TABLE 2:** Interaction between MeDi and overall rsFC change on change in performance on each reference ability.

		MeDi group comparisons for interaction with rsFC Δ			
	Interaction between MeDi group and rsFC Δ	Moderate vs. low MeDi		High vs. low MeDi	
	*p*	B (LL, UL)	*p*	B (LL, UL)	*p*
**MEMORY**					
Between	0.033*	4.182 (−0.047, 8.412)	0.053^†^	6.696 (1.598, 11.795)	0.010*
Within	0.028*	3.837 (0.193, 7.482)	0.039*	5.384 (1.242, 9.526)	0.011*
**FLUID**					
Between	0.643	0.195 (−0.433, 0.824)	0.543	−0.077 (−0.835, 0.680)	0.841
Within	0.871	0.075 (−0.469, 0.619)	0.787	−0.073 (−0.691, 0.545)	0.817
**SPEED**					
Between	0.717	0.213 (−0.637, 1.062)	0.623	−0.115 (−1.140, 0.909)	0.825
Within	0.926	0.131 (−0.603, 0.866)	0.726	0.018 (−0.817, 0.853)	0.966
**VOCAB**					
Between	0.347	0.628 (−0.246, 1.503)	0.159	0.617 (−0.437, 1.672)	0.251
Within	0.697	0.331 (−0.434, 1.096)	0.397	0.233 (−0.637, 1.102)	0.600

*p < 0.05; ^†^*p* < 0.10. B = unstandardized regression coefficient, with 95% Wald confidence intervals (LL, limit; UL, upper limit). Δ, change from baseline to followup; FLUID, fluid reasoning; MEMORY, episodic memory; VOCAB, vocabulary; SPEED, perceptual speed.

**TABLE 3:** Effect of change in rsFC by network on change in memory performance within each MeDi group.

	Effects of rsFC Δ on MEMORY Δ within MeDi groups					
	Low MeDi		Moderate MeDi		High MeDi	
Network	B (LL, UL)	*p*	B (LL, UL)	*p*	B (LL, UL)	*p*
**BETWEEN NETWORK**						
All	−4.397 (−8.269, −0.524)	0.026*	−0.751 (−3.136, 1.63)	0.537	3.287 (0.823, 5.750)	0.009**
Hand	−1.897 (−4.924, 1.130)	0.219	−0.616 (−2.427, 1.196)	0.505	2.897 (0.917, 4.878)	0.004**
Vis	−4.929 (−8.583, −1.275)	0.008**	−0.791 (−3.112, 1.530)	0.504	1.583 (−0.521, 3.687)	0.140
Mouth	−3.010 (−5.609, −0.411)	0.023*	−1.491, (−4.127, 1.146)	0.268	1.032 (−0.760, 2.823)	0.259
Aud	−3.546 (−6.408, −0.684)	0.015*	−0.660 (−2.961, 1.641)	0.574	2.667 (0.859,4.475)	0.004**
DMN	−3.658 (−7.601, 0.285)	0.069^†^	−0.609 (−3.076, 1.857)	0.628	2.553 (0.204, 4.902)	0.033*
FP	−3.125 (−6.566, 0.315)	0.075^†^	−0.200 (−2.451, 2.050)	0.862	3.048 (0.941, 5.156)	0.005**
VAN	−0.197 (−4.676, 4.282)	0.931	−0.415 (−2.403, 1.573)	0.683	0.914 (−1.268, 3.096)	0.412
CO	−3.220 (−5.634, −0.807)	0.009**	−0.720 (−2.940, 1.500)	0.525	1.836 (−0.591, 4.263)	0.138
DAN	−2.314 (−5.550, −0.923)	0.161	−0.945 (−3.243, 1.353)	0.420	1.605 (−0.425, 3.635)	0.121
Sal	−3.980 (−7.427, −0.533)	0.024*	−1.414 (−4.458,1.629)	0.362	3.657 (1.324, 5.990)	0.002**
**WITHIN NETWORK**						
All	−4.866 (−8.010, −1.723)	0.002**	−0.1.267 (−3.705, 1.170)	0.308	2.564 (0.252, 4.876)	0.030*
Hand	1.032 (−0.850, 2.914)	0.283	−0.105 (−1.461, 1.250)	0.879	0.900 (−0.396, 2.197)	0.173
Vis	−0.856 (−2.503, 0.790)	0.308	−0.175 (−1.483, 1.133)	0.794	1.384 (0.629, 2.138)	<0.001***
Mouth	1.098 (−0.325, 2.521)	0.131	0.395 (−0.751, 1.540)	0.500	−0.613 (−1.660, 0.434)	0.251
Aud	−1.435 (−3.046, 0.175)	0.081^†^	0.0163 (−1.163, 1.489)	0.856	1.100 (0.045, 2.174)	0.041*
DMN	−3.534 (−5.452, −1.616	<0.001***	−1.912 (−3.986, 0.161)	0.071^†^	−0.521 (−2.050, 1.009)	0.505
FP	−3.265 (−5.870, −0.659)	0.014*	2.678 (0.534, 4.821)	0.014*	0.218 (−0.945, 1.381)	0.120
VAN	−1.638 (−3.136, −0.140)	0.032*	0.201 (−1.696, 2.097)	0.836	−0.256 (−1.208, 0.697)	0.599
CO	−1.014 (−2.717, 0.690)	0.243	−1.178 (−2.772, 0.367)	0.135	1.840 (0.745, 2.936)	<0.001***
DAN	0.282 (−1.351, 1.914)	0.735	1.106 (−1.089, 3.301)	0.323	0.382 (−0.619, 1.382)	0.454
Sal	−1.378 (−3.378, 0.623)	0.177	−0.071 (−1.951,1.812)	0.941	0.898 (−0.793, 2.590)	0.298

^†^*p* < 0.10; *p < 0.05; **p < 0.01; ***p < 0.001. B = unstandardized regression coefficient, with 95% Wald confidence intervals (LL, limit; UL, upper limit). Δ, change from baseline to followup; FLUID, fluid reasoning All = overall inter-network rsFC; Vis, visual; Aud, auditory; DMN, default mode network; FP, fronto-parietal; VAN, ventral attention network; CO, cingulo-opercular; DAN, dorsal attention network; Sal, salience.

### Interaction between MeDi and change in overall within-network rsFC on cognition

**FIGURE 2:**
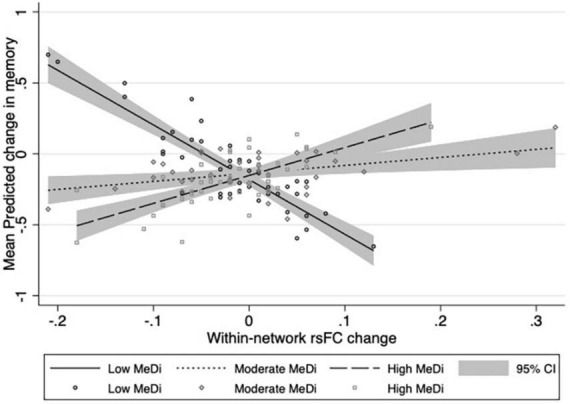
Relationship between change in overall within-network rsFC and change in memory performance by MeDi group. Results of multivariable linear regressions modeling interaction between MeDi group and change in overall within-network rsFC on change in memory performance. Models adjusted for effects of age, gender, race, IQ, caloric intake, mean cortical thickness, diffusion tensor imaging fractional anisotropy, white matter hyperintensities, total brain volume, and scrub percentage. CI, confidence intervals.

For MEMORY, we found a significant interaction between MeDi group and overall within-network rsFC change on change in performance (*p-interaction* = 0.028): relative to the low MeDi group, the association between within-network rsFC change and change in MEMORY was weaker in the moderate MeDi group (*p-interaction* = 0.039) and high MeDi group (*p-interaction* = 0.011) (Table 2 and Figure 2). Analyses of the effect of within-network rsFC change on MEMORY change stratified by MeDi group demonstrated that there was a significant negative effect of change in rsFC on change in MEMORY for the low MeDi group (*B* = −4.866 [−8.010, −1.723], *p* = 0.002), no significant relationship in the moderate MeDi group, and a significant positive effect of change in rsFC on change in MEMORY in the high MeDi group (*B* = 2.564 [0.252, 4.876], *p* = 0.030). MeDi did not moderate the effect of change in overall within-network rsFC on change in FLUID (*p-interaction* = 0.811), SPEED (*p-interaction* = 0.896), or VOCAB (*p-interaction* = 0.663) performance.

### Exploratory analyses

#### Average between-network and within-network rsFC for each network

In exploratory analyses of between-network rsFC for each individual network, we found significant or marginally significant effects of MeDi on the association between change in rsFC and change in MEMORY for Visual (*p* = 0.023), Auditory (*p* = 0.022), CO (*p* = 0.054), DAN (*p* = 0.098), and Salience (*p* = 0.011) networks (Supplementary Table 2). Analyses stratified by MeDi group showed that for nearly all networks, change in between-network rsFC was significantly negatively associated with change in MEMORY in the low MeDi group, and significantly positively associated with MEMORY change in the high MeDi group (Table 3).

In analyses of within-network rsFC for each network, we found that the association between change in within-network rsFC and change in MEMORY significantly differed by MeDi group for the FP network (*p* = 0.010; Supplementary Table 2). Despite the interaction between MeDi and within-network rsFC not reaching statistical significance for other individual networks, analyses stratified by MeDi group showed that in nearly all networks, decrease in within-network rsFC predicted greater positive change in MEMORY for the low MeDi group, whereas in the high MeDi group, increased within-network rsFC predicted greater increase in MEMORY performance (Table 3).

#### Relationship between MeDi, rsFC change and memory change stratified by age

To test whether the moderating effect of MeDi on the associations between change in rsFC and change in MEMORY for each network differed by age, we conducted multivariable linear regressions, controlling for all covariates described above, stratified by age groups. For both between-network and within-network rsFC, the interaction between MeDi, overall rsFC change, and memory change was significant for older adults but not for younger or middle-aged adults (*p* < 0.001 for both; Table 4). For between-network rsFC, this effect was seen in older adults in eight of the 10 networks of interest. For within-network rsFC, four of the 10 networks showed a significant or marginally significant interaction in the oldest age group, and there was a significant interaction in the middle-aged group for two networks, and in the youngest age group for two networks (Table 4).

**TABLE 4:** Interactions between MeDi and change in rsFC on change in memory performance for each network, stratified by age group.

	Interaction between MeDi group and rsFC Δ on MEMORY Δ within age groups					
	Younger adult		Middle age		Older adult	
Network	Wald-chi square	*p*	Wald-chi square	*p*	Wald-chi square	*p*
**BETWEEN**						
All	0.040	0.980	0.814	0.666	14.939	<0.001***
Hand	0.805	0.669	2.149	0.341	3.290	0.193
Vis	0.103	0.950	1.066	0.587	16.284	<0.001***
Mouth	0.759	0.684	0.404	0.817	1.277	0.528
Aud	3.863	0.145	1.852	0.396	14.265	<0.001***
DMN	0.074	0.964	0.635	0.728	8.063	0.018*
FP	0.401	0.818	0.031	0.984	13.422	0.001**
VAN	1.232	0.540	5.928	0.052^†^	7.897	0.019*
CO	1.861	0.392	0.334	0.846	18.739	<0.001***
DAN	0.937	0.626	0.853	0.653	6.142	0.046*
Sal	1.984	0.371	1.310	0.519	5.313	0.070^†^
**WITHIN**						
All	2.652	0.266	1.788	0.409	18.526	<0.001***
Hand	0.882	0.643	0.708	0.702	0.790	0.674
Vis	1.475	0.478	0.141	0.932	5.865	0.053^†^
Mouth	5.770	0.056^†^	1.372	0.504	0.683	0.711
Aud	2.971	0.226	1.414	0.493	6.429	0.040*
DMN	5.191	0.075^†^	3.021	0.221	7.421	0.024*
FP	12.930	0.002**	2.227	0.328	3.586	0.166
VAN	7.139	0.028*	6.541	0.038*	7.087	0.029*
CO	0.663	0.718	8.190	0.017*	4.294	0.117
DAN	1.527	0.466	1.444	0.486	3.615	0.164
Sal	1.815	0.403	1.522	0.467	2.174	0.337

^†^*p* < 0.10; *p < 0.05; **p < 0.01; ***p < 0.001. Δ, change from baseline to followup; Vis, visual; Aud, auditory; DMN, default mode network; FP, fronto-parietal; VAN, ventral attention network; CO, cingulo-opercular; DAN, dorsal attention network; Sal, salience.

Sensitivity analyses were also conducted to examine whether MeDi moderated associations between changes in overall rsFC and changes in cognition when MeDi scores were modeled as a continuous rather than categorical variable. Results showed that continuous MeDi score remained a marginally significant moderator of the relationship between change in overall between-network rsFC on change in memory (*B* = 0.405 [−0.047, 0.857]; *p* = 0.079) and between change in overall within-network rsFC on change in memory (*B* = 0.332, [−0.033, 0.697]; *p* = 0.075), with no significant effects for other cognitive domains (all *p* > 0.34).

## Discussion

In the current longitudinal population-based study, we found that adherence to MeDi significantly moderated the relationship between change in both between- and within-network rsFC and change in memory performance over an average of 5 years. To our knowledge, this is the first study to test the relationship among MeDi, change in connectivity at rest, and change in cognitive performance, and demonstrate that MeDi may benefit cognition by mitigating the negative effect of altered connectivity on cognitive function over time.

Of the four RAs tested, MeDi had the most robust effect on the relationship between rsFC and memory. This is consistent with past research suggesting episodic memory is among the cognitive domains most influenced by effects of both normal aging and neuropathological change (Small, 2001; Deary et al., 2009; Correia et al., 2018). The finding that the interaction between MeDi and change in between-network rsFC was present for nearly all individual networks, whereas effects for within-network connectivity were limited to specific networks, is also consistent with existing research: studies have shown that between-network connectivity may be more predictive of changes in memory performance, whereas connectivity within different individual networks may differentially predict performance across different cognitive domains (Chan et al., 2014; Varangis et al., 2019b; Stumme et al., 2020).

A considerable amount of past research has also suggested that within-network rsFC tends to decrease with age, and that these decreases in connectivity often predict decreases in cognitive function (Betzel et al., 2014; Geerligs et al., 2014; Song et al., 2014; Varangis et al., 2019a). In contrast, the current sample demonstrated both increases and decreases in within-network rsFC over 5 years, and decreases in rsFC were associated with increases in memory performance. However, our findings are consistent with research showing that for some networks— including frontoparietal network, in which we found significant effects— age is associated with increases in within-network rsFC, which predict memory decline (Sala-Llonch et al., 2014; Jockwitz et al., 2017). These changes are thought to reflect age-related compensatory increases in connectivity within relevant networks in an attempt to maintain cognitive performance. Interpreting our results in the context of these findings, increases in frontoparietal rsFC may be associated with decreases in performance in the low MeDi group due to compensatory processes, whereas individuals in moderate and high MeDi groups may be better able to maintain performance without compensatory increases in frontoparietal connectivity.

The mechanisms by which MeDi protects against negative cognitive effects of neurological change remain unclear, but past research has suggested foods comprising MeDi may reduce inflammation and oxidative stress (Siervo et al., 2021). MeDi may also protect against chronic diseases, such as cardiovascular disease and diabetes (Wu and Sun, 2017), and may therefore indirectly protect neurocognitive function by reducing the risk of other diseases known to have detrimental effects on brain and cognitive health.

To our knowledge, this is the first longitudinal study to examine the effects of dietary patterns on changes in within- and between-network rsFC, and to evaluate the moderating effect of MeDi on the relationship between changes in rsFC and change in cognitive performance over time. One strength of the current study is the inclusion of adults across a broad age range, in contrast to past literature that has primarily focused on the effects of lifestyle factors on brain and cognitive function in only older adult populations. Our exploratory analyses revealed that older adults showed a greater protective effect of MeDi on the negative cognitive effects of changes in rsFC, suggesting the impact of MeDi may be most beneficial in mitigating age-related neurocognitive decline. The current study is also strengthened by adjusting for the potential confounding effects of structural brain measures on change in rsFC and cognition, which allows us to interpret a direct role of diet on the relationship between connectivity and performance above and beyond structural brain health.

The small amount of existing research on the role of diet in neurocognitive change has primarily focused on the effects of individual nutrients, the interpretation of which is limited by the fact that typical diets are composed of multiple potentially interacting and inter-correlated nutrients (Scarmeas et al., 2009; Gu and Scarmeas, 2011). Moreover, these studies tend to evaluate the effects of short-term consumption of nutrients, which fails to capture the cumulative effects of habitual dietary patterns. Therefore, a significant strength of the current study is the use of a comprehensive dietary measure that reflects habitual intake of multiple food types, which significantly contributes to our understanding of the neurocognitive effects of long-term dietary patterns.

One potential limitation of the current study is the use of composite cognitive ability scores based on both in-scanner tasks and out-of-scanner neuropsychological assessments. As such, our results may not directly support or contradict findings in prior research on the relationships between rsFC, MeDi, and specific cognitive processes, but rather, provide important evidence that MeDi moderates the relationship between rsFC changes and changes in broad cognitive domains. Due to limited sample size, we also did not correct for multiple comparisons in our statistical analyses, resulting in the potential for type 1 errors. However, the consistency of our results across nearly all networks of interest suggests a lower likelihood of false-positive results, and these findings are also consistent with our past work showing MeDi moderates the relationship between rsFC and cognitive function in a cross-sectional sample (Gaynor et al., 2022). As such, these preliminary findings provide encouraging evidence of a relationship between MeDi, longitudinal change in rsFC and change in cognition that warrant further investigation in future studies with larger sample sizes.

Another potential limitation is the use of external parcelation scheme to define resting state networks. Although it is possible to base network parcelation on participants’ own network structures, the wide age range of participants in the current sample could have introduced age-related biases in network assignment. Moreover, previous work from our group and others, including our recent cross-sectional study of the interaction between MeDi and between-network rsFC on cognition (Gaynor et al., 2022), has utilized the current network taxonomy;(Song et al., 2014; Varangis et al., 2019a,b) as such, the use of a common parcelation scheme allows us to more accurately interpret the current results in the context of our previous work, as well as allowing for more accurate reproducibility of these results in other cohorts. It is also important to note that we evaluated overall within- and between-network connectivity using a single value reflecting average correlations among all 10 networks of interest, but this measure may not fully capture the complexities of network dynamics and how they change over time. Given that this is the first study to date to test effects of MeDi in the context of functional brain measures and cognition over time, we used a parsimonious approach to model the effect of MeDi on broad measures of brain and cognitive function; in light of our significant preliminary findings, further research is warranted to understand how diet interacts with changes in individual networks, and with more precise measures of nodal and global connectivity and efficiency. Although we focused on overall positive correlations between resting state network activity, it is also important to note that some individual networks have been shown be negatively correlated, such as well-known negative associations between activity in the DMN and task-related functional networks (Greicius et al., 2003; Fox et al., 2005). We chose to exclude negatively correlated ROIs between networks based on our group’s past research showing that compared to positive correlations, negative between-network rsFC was less consistently related to both age and cognitive function in the same sample (Varangis et al., 2019a), and our recent findings that MeDi moderated the effect of positive between-network rsFC on cognition in a cross-sectional sample (Gaynor et al., 2022). Future research aimed at clarifying the role of MeDi on individual resting-state networks would benefit from the analysis of negative correlations between individual networks known to work in opposition.

## Conclusion

Results of the current study suggest that MeDi may support cognitive function despite changes in brain connectivity, as evidenced by an attenuated effect of change in connectivity on change in cognitive function in those with higher MeDi adherence. Future research is warranted to understand the mechanisms by which MeDi exerts its neuroprotective effects, as well as the role of other modifiable lifestyle factors in the relationship between brain biomarkers and cognitive function over the lifespan.

## Data availability statement

The raw data supporting the conclusions of this article will be made available by the authors, without undue reservation.

## Ethics statement

The studies involving human participants were reviewed and approved by the Institutional Review Board of the College of Physicians and Surgeons of Columbia University. The patients/participants provided their written informed consent to participate in this study.

## Author contributions

YGu and AG: conception and design of the work and drafting of the manuscript. All authors: critical revision and final approval of the manuscript. All authors fulfill the ICMJE criteria for authorship.
