# Supplementary material for: Longitudinal association between changes in resting-state network connectivity and cognition trajectories: The moderation role of a healthy diet
Source: Front Hum Neurosci. 2023 Jan 19;16:1043423. doi: 10.3389/fnhum.2022.1043423 (PMC9893792; doi:10.3389/fnhum.2022.1043423)
Supplement: Supplementary file 1 [file Data_Sheet_1.pdf]

Supplementary Table S1. Scanning parameters for MRI sequences.

Sequence	Parameters
GE EPI	TE/TR: 20/2000 ms; Field of view: 240mm; Flip angle: 72°; In-plane resolution: 112×112; Slice thickness/gap: 3/0 mm; Slices: 37; volumes: 285
MPRAGE	TE/TR: 3/6.5 ms; Field of view: 256 mm; Flip angle: 8°; In-plane resolution: 256x256; Slice thickness/gap: 1/0 mm; Slices: 180
DTI	55 directions; b: 800 s/mm2; TE/TR: 69/11032 ms; Flip angle: 90°; In-plane resolution 112x112; Voxels: 2x2x2 mm; Slices: 75
FLAIR	TE/TR: 2800/11000 ms; Field of view: 23.0x17.96 cm; In-plane resolution: 256x189; Slice thickness/gap: 4/0.5 mm; Slices: 30

Abbreviations: GE EPI= gradient-echo echo-planar imaging; MPRAGE= magnetization-prepared rapid gradient-echo; DTI= diffusion tensor imaging; FLAIR= fluid-attenuated inversion recovery; TE= echo time; TR= repetition time.
