# Supplementary material for: Longitudinal association between changes in resting-state network connectivity and cognition trajectories: The moderation role of a healthy diet
Source: Front Hum Neurosci. 2023 Jan 19;16:1043423. doi: 10.3389/fnhum.2022.1043423 (PMC9893792; doi:10.3389/fnhum.2022.1043423)
Supplement: Supplementary file 2 [file Data_Sheet_2.PDF]

Supplementary Table S2. Interactions between effects of MeDi group and change in rsFC between each network and all others on memory performance

		Parameter estimates for rsFC*MeDi group comparisons			
	rsFC * MeDi	Moderate vs. Low MeDi		High vs. Low MeDi	
	p	B [LL, UL]	*p*	B [LL, UL]	*p*
BETWEEN					
All	.033*	4.182 [-0.047, 8.412]	.053†	6.696 [1.598, 11.795]	.010*
Hand	.103	1.784 [-1.370, 4.938]	.268	4.203 [0.326, 8.081]	.034*
Vis	.023*	4.769 [0.765, 8.772]	.020*	6.219 [1.614, 10.824]	.008**
Mouth	.559	1.410 [-1.814, 4.633]	.391	1.681 [-1.661, 5.024]	.324
Aud	.022*	3.431 [0.048, [6.814]	.047*	5.245 [1.463, 9.028]	.007**
DMN	.126	3.432 [-0.849, 7.713]	.116	5.157 [0.056, 10.258]	.048*
FP	.101	3.420 [-0.545, 7.386]	.091†	4.788 [0.249, 9.328]	.039*
VAN	.840	0.876 [-3.741, 5.492]	.710	1.606 [-3.764, 6.976]	.558
CO	.054†	2.923 [-0.226, 6.072]	.069†	4.567 [0.491, 8.644]	.028*
DAN	.098†	2.451 [-1.254, 6.156]	.195	4.537 [0.411, 8.663]	.031*
Sal	.011*	3.224 [-1.034, 7.483]	.138	7.094 [2.468, 11.720]	.003**
WITHIN					
All	.028*	3.837 [0.193, 7.482]	.039*	5.384 [1.242, 9.562]	.011*
Hand	.798	-0.411 [-2.557, 1.735]	.708	-0.855 [-3.291, 1.582]	.492
Vis	.210	0.528 [-1.336, 2.391]	.597]	1.609 [-0.255, 3.474]	.091†
Mouth	.432	-0.454 [-2.123, 1.215]	.594	-1.279 [-3.214,0.657]	.195
Aud	.180	1.609 [-0.402, 3.621]	.117	1.890 [-0.340, 4.120]	.097†
DMN	.104	2.347 [-0.296, 4.991]	.082†	2.589 [-0.081, 5.260]	.057†
FP	.010*	4.697 [1.662, 7.732]	.002**	2.621 [-0.106, 5.348]	.060†
VAN	.646	1.032 [-1.247, 3.310]	.375	0.635 [-1.372, 2.641]	.535
CO	.124	0.059 [-2.075, 2.193]	.957	2.180 [-0.134, 4.495]	.065†
DAN	.634	1.099 [-1.209, 3.407]	.351	0.660 [-1.433, 2.754]	.536
Sal	.184	1.475 [-1.162, 4.112]	.273	2.712 [-0.238, 5.662]	.072†

†*p*<.10; **p*<.05; ***p*<.01

B= unstandardized regression coefficient, with 95% Wald confidence intervals [LL: limit, UL: upper limit].

Abbreviations: All= overall internetwork rsFC; Vis= Visual; Aud= Auditory; DMN= default mode network; FP= fronto-parietal; VAN= ventral attention network; CO= cingulo-opercular; DAN= dorsal attention network; Sal= salience; FLUID= fluid reasoning; MEMORY= episodic memory; VOCAB= vocabulary; SPEED= perceptual speed.
